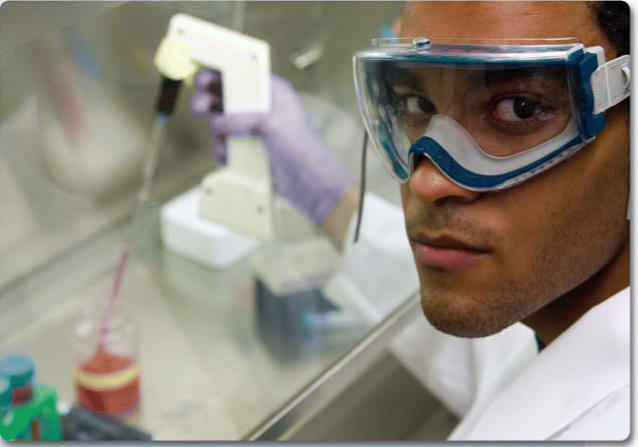# Research Opportunities for College Undergraduates and High School Students

**Published:** 2006-12

As part of the Strategic Plan, the National Institute of Environmental Health Sciences has announced two new initiatives to enhance opportunities for young, motivated high school and undergraduate students to participate actively in research and to increase the visibility of the field of environmental health sciences.

The NIEHS Short Term Educational Experiences for Research (STEER) in the Environmental Health Sciences for Undergraduates and High School Students (R25) seeks research institutions that will create and provide innovative summer research and educational opportunities in the environmental health sciences for up to eight talented high school students and college undergraduates.

The R25 short-term educational programs will have an organized summer program of student research experiences with participating faculty in the environmental health sciences, and a program of informational exchange designed to convey to student participants an appreciation of research on the environmental impacts on human health. The focus of both the laboratory experience and the educational experiences/seminars will be on human health outcomes related to environmental exposure. These programs must have a sufficient base of NIEHS and NIH environmental health–related research to justify their proposed number of student participants.

The NIEHS also announces an administrative supplement program available to principal investigators with R01, R37, or P01 awards to support individual summer research experiences for talented and gifted high school students and college undergraduates. Any NIEHS-supported R01, R37, or P01 with a minimum of 1 year of funding remaining at the time of submission is eligible for this supplement program. A currently funded principal investigator may have only one student investigator funded through this supplement program at any given time.

For more information on these programs see:

STEER: http://grants.nih.gov/grants/guide/rfa-files/RFA-ES-06-009.html

Supplements: http://www.niehs.nih.gov/dert/training/HScolsup.htm

## Contacts

**Michael C. Humble, Ph.D.** | humble@niehs.nih.gov

## Figures and Tables

**Figure:**